# Investigation of varenicline and tropisetron in latent inhibition and novel object recognition in mice

**DOI:** 10.1038/s41598-026-41544-w

**Published:** 2026-03-03

**Authors:** Lorena R. Lizarraga-Valderrama, Stuart Williams, David J. G. Watson, Kiri T. Granger, Claire L. Gibson, Paula M. Moran

**Affiliations:** 1 School of Psychology, University of Nottingham, University Park Campus, Nottingham, NG72RD UK; 2 Monument Therapeutics Ltd., Alderley Park, Congleton Road, Macclesfield, SK10 4TG UK; 3 School of Biosciences, Cardiff University, Cardiff, CF103AX UK; 4 School of Science and Technology, Nottingham Trent University, Clifton Campus NG118NS, Nottingham, UK; 5 School of Life Sciences, Queen’s Medical Centre, The University of Nottingham, Nottingham, NG7 2UH UK

**Keywords:** Drug discovery, Neuroscience, Psychology, Medical research

## Abstract

There is currently no treatment for cognitive-impairment associated with schizophrenia (CIAS). Animal and human studies suggest that nicotinic cholinergic agonists and 5-HT3 antagonists have potential therapeutic benefits. We investigated whether co-administration of varenicline, an alpha4beta2 nAChR partial agonist and alpha7 nicotinic receptor agonist, and tropisetron, a 5-HT3 antagonist and alpha7 nicotinic receptor agonist is pro-cognitive in male c57/Bl6 mice. We first assessed Latent inhibition (LI) an associative learning process of learning to ignore irrelevant stimuli. We report no effect of tropisetron on LI (0.01, 0.04, 0.4 mg/kg s.c.). Varenicline at 0.5 mg/kg s.c. potentiated LI. Co-administration of tropisetron 0.4 mg/kg s.c. and 0.5 mg/kg s.c. varenicline abolished varenicline’s potentiation of LI. In 24 h retention of novel object recognition (NOR) where memory performance (discrimination-index) is poor in controls there was no effect of varenicline or tropisetron. When varenicline (0.5 mg/kg) and tropisetron (0.01, 0.1, 1.0 mg/kg) were co-administered NOR discrimination-index was higher compared to controls. These data indicate that varenicline enhances LI when low in controls, demonstrate an interaction between varenicline and tropisetron in LI and suggest synergistic interaction between tropisetron and varenicline to potentiate NOR memory merits further investigation. These findings have implications for treatment of CIAS and disorders with cognitive impairment.

**Supplementary Information:**

The online version contains supplementary material available at 10.1038/s41598-026-41544-w.

## Introduction

Cognitive impairment associated with schizophrenia (CIAS) is a core psychopathological symptom of the illness. Impairments can be moderate to severe affecting several cognitive domains^1,2^, including general intelligence, vigilance/attention^3^, working memory^4^, verbal learning and memory^5^, executive functions^6^, reasoning and problem-solving, processing speed, and social cognition^7^. Treatments for CIAS are urgently needed as it is a strong predictor of functional outcomes for the disease^8,9^. Despite the global burden associated with these cognitive deficits, this aspect of the disorder has been largely neglected. Currently there is no specific treatment available^10^. Considerable effort has been devoted to developing antipsychotic drugs, but these are minimally if at all ineffective in reducing cognitive impairments^11^. One approach suggested to address CIAS has been to target nicotinic cholinergic receptors (nAChRs). The most predominant nAChRs subtypes in the brain are the heteropentameric α4β2 nAChRs and the homopentameric α7 nAChRs^12,13^. Both of these nAChRs are involved in cognitive functioning and in the pathophysiology of schizophrenia, most studies have focused on α7 nAChRs. Both, α7 nAChRs and α4β2 nAChRs are highly expressed in brain areas involved in cognition. For instance, α7 nAChR subtype is highly enriched in the hippocampus, cortex, and subcortical limbic regions^14^ while α4β2 nAChRs are located in the cortex, hippocampus, and thalamus^15^.

There is a wealth of evidence associating nicotinic receptors and schizophrenia (see^16^ for review). There is genetic evidence showing SNPs in the core promoter of the α7 nAChR gene *CHRNA7* have been associated with reduced expression of α7 nAChRs and an increased risk for schizophrenia^17^. Post-mortem studies of patients with schizophrenia who smoke have shown that high affinity α4β2 nAChR binding of [3 H]-nicotine and [3 H]-epibatidine is reduced in the hippocampus, cortex, and caudate^18^. Decreased protein level of α7 nicotinic receptor has been shown in frontal cortex of patients with schizophrenia^19^. In vivo single-photon emission computed tomography (SPECT) has shown a correlation between upregulated β2 nAChR and executive function in patients^20^. The α7 nAChR selective radioligand 18 F-ASEM binding has been shown to be lower in patients with schizophrenia compared to healthy volunteers in cingulate and frontal cortex, and hippocampus^21,22^. Moreover, in recent onset schizophrenia ligand binding was found to correlate with composite score on a global cognitive test battery that measured processing speed, attention, auditory verbal and visuospatial memory executive function and ideational fluency^21^.

Extensive preclinical evidence shows that α7 nAChR ligands have pro-cognitive effects. Notably, several α7 nAChR agonists have been shown to improve cognitive deficits considered to be schizophrenia relevant in rodent models^23–32^. These studies provide evidence of restoration of cognitive impairments in various domains, including attentional set-shifting, memory formation and retrieval, and sensorimotor gating deficits^33^. α4β2 AChR agonists have also been shown to improve various cognitive deficits in animal models^15^ including attention, working memory and visual memory^34–36^.

The potential of nicotinic agonists indicated by preclinical studies is supported by clinical evidence. Chronic use of varenicline, a nAChR agonist, enhances cognitive function and working memory in abstinent smokers^37,38^. Varenicline is a full agonist of α7 nAChRs and partial agonist of α4β2 nAChR and 5-HT3^39^ and is currently used for smoking cessation and for the treatment of dry eye disease. Studies in animal models of cognitive impairment have revealed that varenicline improves recognition memory suggesting that it may be effective to treat CIAS^40,41^. Other classes of drugs have partial agonist properties at nicotinic receptors. Tropisetron, a 5-HT3 antagonist also exhibits high affinity, partial agonist activity at α7 nAChRs, and has shown to enhance recognition memory in rats^42,43^. Several studies in patients with schizophrenia with tropisetron have shown improvements in cognitive processes including auditory sensory gating and rapid visual information processing and immediate memory^44–46^. One impediment to development of tropisetron for CIAS was gastrointestinal side effects consequent to 5-HT3 antagonism^47,48^. Varenicline in addition to its nicotinic action also has 5-HT3 agonist properties^49^. Varenicline is associated with nausea as a side effect raising the possibility that co-administration with a 5-HT3 antagonist might alleviate this effect. With a view to future studies evaluating the combination of varenicline and tropisetron in human studies for CIAS, we aimed to investigate the pro-cognitive properties of varenicline and tropisetron and to ascertain whether their combination produced synergistic activity on cognition in mice.

We first investigated whether tropisetron potentiates latent inhibition. Latent inhibition (LI) is a measure of the ability to learn to ignore irrelevant stimuli and is disrupted in individuals with acute schizophrenia and sensitive to antipsychotic drugs^50–52^. LI has been extensively studied in various species, including humans, because of its relevance in learning and selective attention, abnormalities observed in patients with schizophrenia, individuals at high risk for schizophrenia and healthy populations who score highly on psychometric measures of schizotypy^51,53–55^. In human and animal studies LI shows pharmacological sensitivity (both disruption and enhancement dependent upon the paradigm) to drugs with known pro and antipsychotic effects^51,52,56–58^ as well as some drugs with putative effects on cognition such as nicotine^51,59,60^. LI is demonstrated as slower learning about a stimulus when it has been pre-exposed without consequence, compared to when it has not been pre-exposed. In animal studies this is frequently a tone or light stimulus and the effect is influenced by the amount of stimulus pre-exposure. LI has been shown to produce different profiles of deficit or enhancement depending upon neurochemical system and experimental conditions manipulated, such as amount of pre-exposure or number of learning trials^57,51^. As such it has been suggested as a potential clinical stratification biomarker to identify subgroups of patients with schizophrenia with glutamatergic, cholinergic and dopaminergic sensitivity^61^. In preclinical studies in mice nicotinic α7 agonist drugs have been shown to potentiate latent inhibition when it is not present in controls^62^.

In a first experiment, tropisetron had no effect on LI. In a second experiment varenicline potentiated LI but when combined with tropisetron, the observed pro-cognitive effect with varenicline alone was reversed, suggesting an interaction between the drugs.

To investigate this further we used an object recognition task with a 24 h retention interval which produces poor memory performance measured as a discrimination index not statistically greater than zero in control mice. The novel object recognition task is commonly used as a preclinical animal model to measure recognition memory and shows selective predictive validity for pro-cognitive drug effects^63–65^.

Neither tropisetron nor varenicline increased discrimination index in object recognition when administered alone, however discrimination index was significantly increased when drugs administered together.

## Results

### Experiment 1 : Effect of tropisetron on latent inhibition

**Fig. 1:**
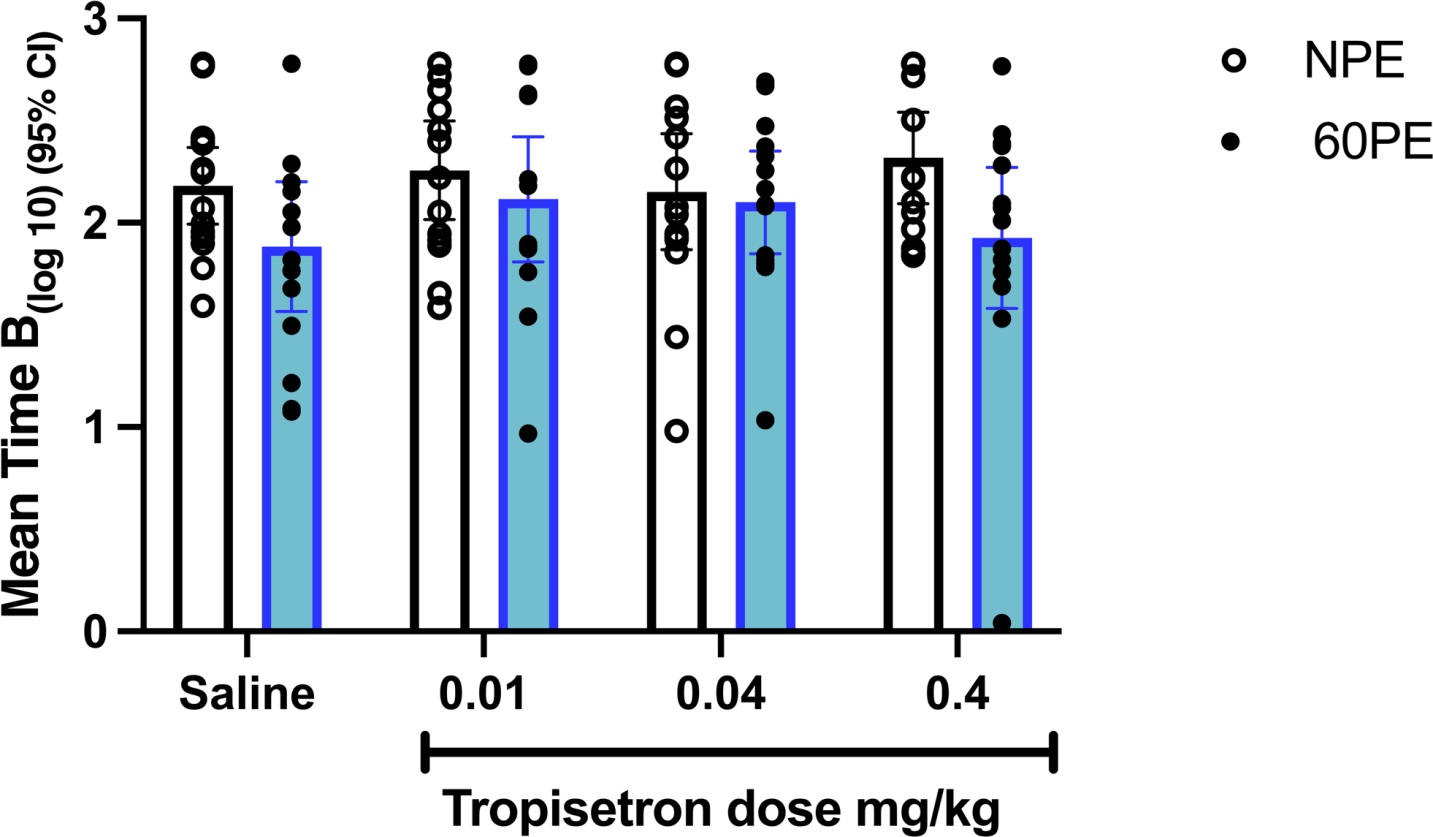
Effect of tropisetron on latent inhibition. Mean (+/− 95% CI) Log 10 Time B (s) i.e., time to complete 10 licks after tone onset. Latent inhibition is higher Log10 time B in NPE vs. PE conditions.

For the Log 10 Time measure there was a significant effect of pre-exposure f (1, 107) = 6.023, *P* = 0.016, ηp² =0.052) but no significant effect of drug group F(3, 107) = 0.489, *P* = 0.691 (Fig. 1). This indicates that tropisetron had no effect on latent inhibition at any of the doses tested. Due to high suppression observed in cohort 1, we additionally conducted further test days for cohorts 2 & 3, this did not alter the conclusion (supplementary section Fig. S1).

### Experiment 2 : Effect of varenicline and varenicline plus tropisetron on latent inhibition

**Fig. 2:**
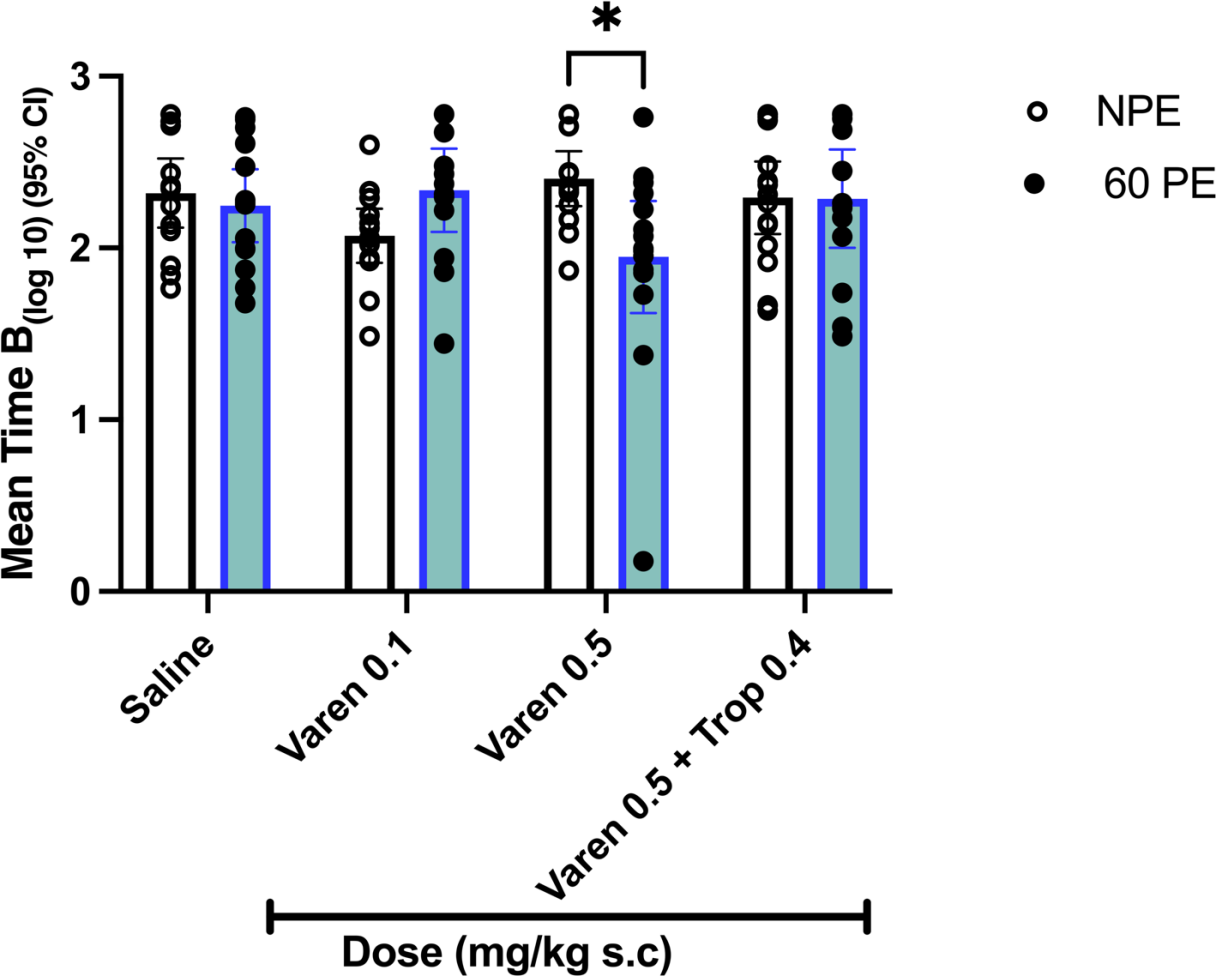
Effect of varenicline and combination varenicline + tropisetron on latent inhibition. mean (+/−  95% CI) Log 10 Time B (s) i.e., time to complete 10 licks after tone onset). Latent inhibition is higher Log10 time B in NPE vs. PE conditions. *indicates *P* < 0.05 significant difference between NPE and PE conditions (i.e., latent inhibition) for varenicline at 0.5 mg/kg.

For the Log 10 Time B measure there was no significant effect of pre-exposure (F (1, 104) = 0.37), nor of drug group (F(3, 104) = 0.634). However a significant interaction between pre-exposure and drug group was observed F(3,104) = 3.82, *P* = 0.01 ηp² =0.099) (Fig. 2). This interaction was followed up by post hoc T-tests with Bonferroni correction. A significant difference between NPE and PE groups was found for varenicline 0.5 mg/kg (T(25) = 3.046, *P* < 0.05) demonstrating the presence of latent inhibition. No such effect was seen for any other treatment conditions (Fig. 2). Although no a priori criteria were set for outlier removal, visual inspection of Fig. 2 suggested the possibility that a single outlier might accentuate the difference between NPE and PE. Reanalysis excluding this outlier [0.28] confirmed a significant NPE Vs PE difference remains in the varenicline 0.5 mg/kg group (*P* < 0.004) and the pre-exposure x drug interaction remained significant F(1,103) = 3.090, *P* = 0.030), ηp² =0.08) .

An additional test day was conducted for both cohorts, replicating the conditions of test day 1. Log 10 Time B again showed a significant interaction between drug and pre-exposure (F(3,108) = 4.446, *P* = 0.006. A significant latent inhibition effect (NPE Vs PE) was observed for tropisetron at 0.5 mg/kg (T(28) = 3.34 *P* < 0.05) (Supplementary section Fig. S2). No other drug condition showed a similar effect. Note that sample sizes differed in these additional test days, as several mice that had not consumed water during re-baselining resumed drinking upon subsequent pre-exposure to the experimental chamber.

### Experiment 3: Effect of tropisetron on novel object recognition

**Fig. 3:**
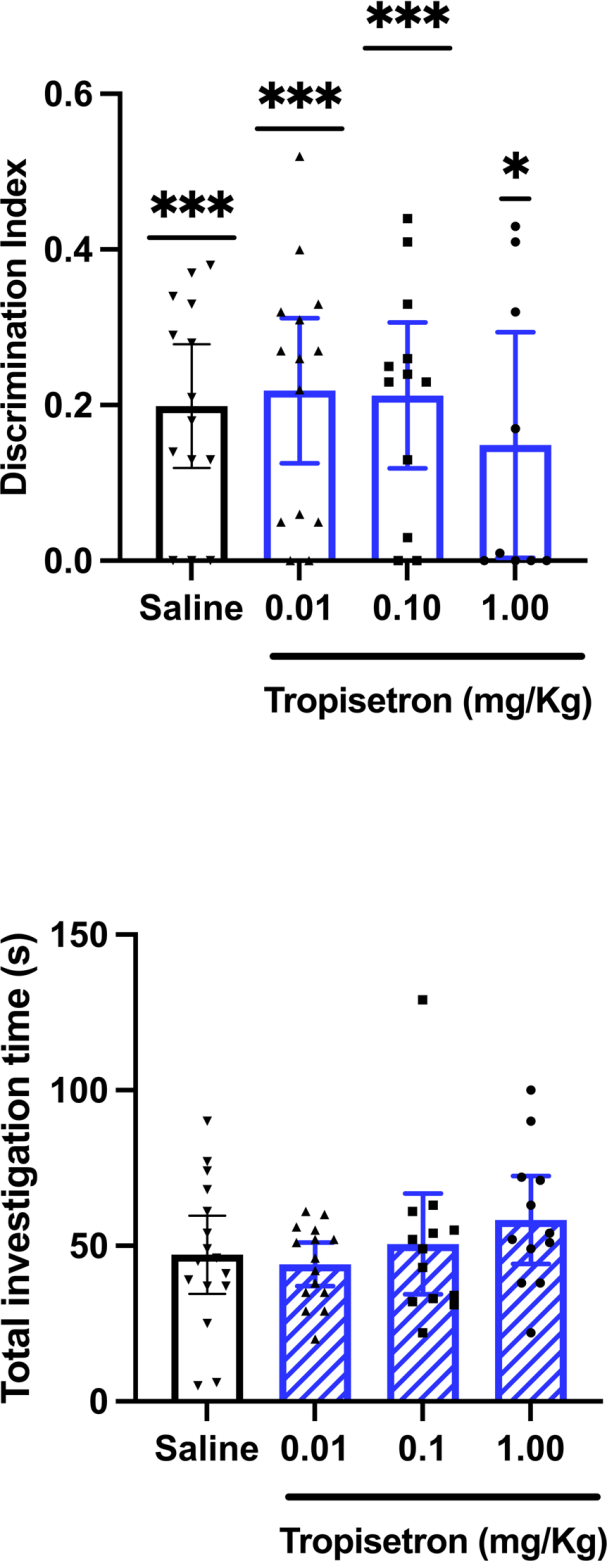
(**A**) Mean (+/−  95% CI) Discrimination index. There were no significant differences between treatment groups. ***P < 0.005, *p < 0.05; significant difference from zero. (**B**) Mean (+/−  95% CI) Total investigation time of objects in sample trial. There were no significant differences between treatment groups.

Discrimination indices were significantly greater than zero for all treatment groups (Fig. 3) indicating successful discrimination of novel from familiar objects in all groups. No significant effect of drug treatment was found on the discrimination index [F(3,45) = 0.4, R^2^ = 0.02] (Fig. 3). Additionally, no significant differences were observed between treatment groups in the time spent investigating objects during the sample trial, suggesting no effect of the drug on overall exploratory behaviour (Fig. 3).There were no significant differences between drug groups in overall activity in either the sample (F < 1) or test trials (F < 1) (supplementary Fig. 6A, B).

### Experiment 4: Effect of varenicline on novel object recognition

**Fig. 4:**
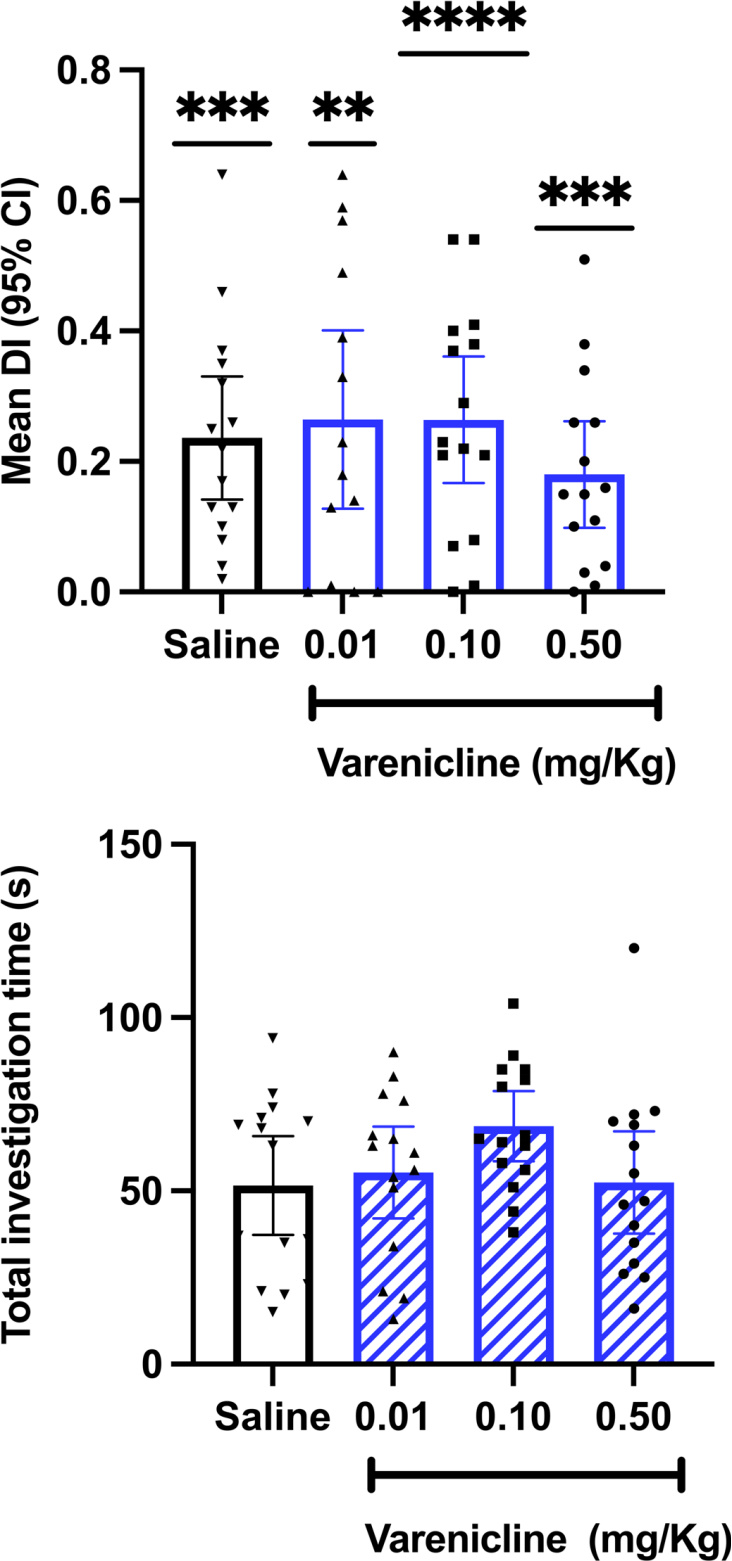
(**A**) Mean (+/−  95% CI) Discrimination index. There were no significant differences between treatment groups. ****P<0.0001, *** *P*<0.0005, **p <0.001; significant difference from zero. (**B**) Mean (+/− 95%CI) Total investigation time of objects in sample trial. There were nosignificant differences between treatment groups.

Discrimination indices were significantly greater than zero for all groups indicating successful discrimination of novel from familiar objects (Fig. 4). No significant effect of drug treatment on discrimination Index was found [F(3,55)] = 0.68, R^2^ = 0.03]. There was a reduction in overall activity in drug treated groups compared to vehicle on the sample trial (F3,48 = 4.10, *P* < 0.01) for 0.01 varenicline (Dunnett’s T=− 2.8, *p* = 0.01) and 0.5 (Dunnett’s T=− 3.32, *p* = 0.01) but no difference on test trials (F < 1) (supplementary Fig. 6C-D). No significant differences between treatment groups were observed in the time spent investigating objects during the sample trial despite this reduced activity suggesting it did not affect equitable exposure to the objects between drug groups (Fig. 4).

### Experiment 5: Effect of varenicline + tropisetron on novel object recognition

**Fig. 5:**
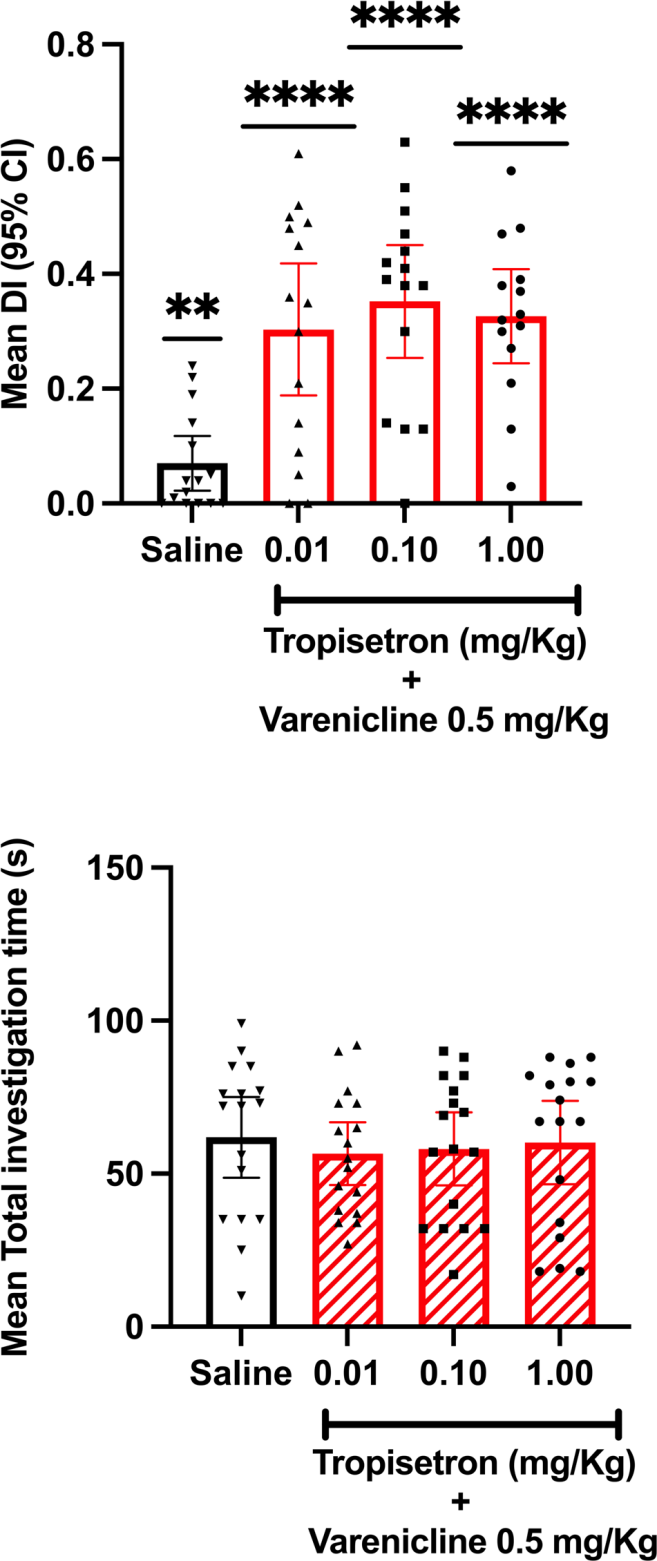
(**A**) Mean (+/−  95% CI) Discrimination Index. ### *P* < 0.0005, ## *P* < 0.001, #### *P* < 0.0001 compared to Saline control group. There was a significant difference from zero. (**B**) Mean (+/−  95% CI) Total investigation time of objects in sample trial. There were no significant differences between treatment groups.

Discrimination indices were significantly greater than zero for all treatment groups (Fig. 5) indicating successful discrimination of novel from familiar objects. A significant effect of drug treatment on discrimination index was observed F(3,55) = 9.84, *P* < 0.0001,R^2^ = 0.349]. Significant differences were found between saline vehicle control and all doses of drug combination on discrimination index using Dunnett’s post hoc test (see Fig. 5). There were no significant differences between drug groups in overall activity in either the sample (F < 1) or test (F3,55 = 1.2) trials (supplementary Fig. 6E-F).

## Discussion

These demonstrate that the nAChR agonist varenicline potentiated latent inhibition while tropisetron potentiated novel object recognition when co-administered with varenicline. In latent inhibition, the pro-cognitive effect of varenicline was reversed by tropisetron. On the other hand, tropisetron and varenicline showed a potential synergistic effect in novel object recognition.

Both 5-HT3 antagonists and nicotinic (partial) agonists have previously been shown to have effects on latent inhibition. Specifically, 5-HT3 antagonists have been reported to potentiate latent inhibition as well as reverse impairments induced by drugs such as D-amphetamine^52,66,67^. Barak et al.^62^ investigated the selective α7 nAChR partial agonist (SSR180711) in models of amphetamine and MK801 latent inhibition disruption. They observed not only reversal of these effects but also unexpectedly noted that SSR180711 enhanced baseline latent inhibition in controls. Our findings support this observation. Using a variant of latent inhibition designed to reveal potentiation if present (i.e., a lower number of tone pre-exposures) we found that varenicline, with a similar mechanism of action to SSR180711, enhanced LI at the higher but not lower dose tested. This suggests the effect may be attributable to its partial agonist activity at α7 nAChR’s. There are several variants of latent inhibition used to model cognitive performance. Latent inhibition can be impaired by pharmacological agents/ lesions/increased shock numbers/intensity or combinations of these and restored by test drugs. Alternatively, it is possible to induce low latent inhibition by reducing the number of pre-exposures and investigating whether it can be restored by a test drug^51,52^. Barak et al. demonstrated that SSR180711 not only reversed impairments induced by glutamate antagonists but also enhanced latent inhibition in unimpaired animals. Both studies show restoration of latent inhibition to “normal “ levels and are therefore consistent with present findings with varenicline. This is also consistent with clinical studies reporting improved cognition with varenicline^68,69^.

Taken together with reports of improved attentional and general cognitive function in humans following varenicline, our data support its potential as a cognitive enhancing drug. These findings also support the potential of latent inhibition to detect α7 nAChR drug activity as has been suggested previously^61^. No effect of tropisetron to enhance latent inhibition distinguishes it from other 5-HT3 antagonist drugs such as dolasetron and ondansetron which have consistently been shown to enhance latent inhibition in rats^66,52^. It is noteworthy that 5-HT3 antagonists differ in their pharmacological profiles, including whether they are competitive or non-competitive and in their action at nicotinic receptors. For example, ondansetron is an α7 nicotinic antagonist^49,70^.

We observed that tropisetron attenuated varenicline-induced LI enhancement when co-administered. This is suggestive of a behavioural interaction between the drugs, as a simple lack of effect of tropisetron would have resulted in an unaltered LI enhancement by varenicline. We cannot determine the mechanism of the observed behavioural interaction from this study but there are several theoretical possibilities. Varenicline is a full agonist at α7 nAChRs and partial agonist at α4β2 nAChR while tropisetron is a partial agonist at α7 nAChRs. It is possible that a hypercholinergic state has induced a hypervigilant or hyperaroused state, which based on findings from fMRI and during cognitive tasks has previously been suggested to heighten sensitivity to irrelevant distractor cues^71^. In latent inhibition such an effect could manifest as greater attention to the pre-exposed cue and consequently moderate varenicline’s enhancement of latent inhibition. This hypothesis warrants further investigation. The behavioural effects of nicotinic agonists including nicotine itself have been well demonstrated to vary depending upon baseline behavioural state. This has been demonstrated in animal studies of reinforcement and locomotor activity as well as cognition^72^. Nicotine agonists including nicotine itself, display an inverted U-shaped dose-response curve with respect to cognitive performance^73,74^. This inverted U-shaped curve is manifested as improved performance at low levels of nicotinic receptor stimulation and impairment at higher doses^75^. It has been suggested that some negative findings in clinical trials with nicotinic drugs such as positive allosteric modulators at nicotinic receptors might be explained by an “overshoot” in terms of receptor stimulation i.e., placing effects at the detrimental portion of the inverted U-shaped curve^76^. The latent inhibition paradigm used in the current study is conducted under conditions of water restriction and involves an aversive conditioning procedure, both of which would be expected to be accompanied by a high arousal state. Non-cholinergic neurotransmitter systems affected by water restriction or aversive conditioning may also be involved in the observed effect of tropisetron on varenicline enhancement of latent inhibition for e.g., dopaminergic transmission in the nucleus accumbens as well as glutamatergic and GABAergic systems which have been shown to play a critical role in mediating LI^51,56,77,78^.

To investigate the possibility of an interaction between varenicline and tropisetron we used a novel object recognition paradigm. This approach allowed us to investigate the generality of the putative effect on cognition. The NOR task is a widely used behavioural assay for studying declarative memory, including both episodic and semantic memory and enables the investigation of distinct stages of recognition memory: encoding, consolidation, and retrieval. In this study, NOR task was employed to assess the effects of tropisetron and varenicline administered before encoding of recognition memory. During memory encoding, the cortex sends sensory inputs to the hippocampus, forming temporary memories. These temporary memories are subsequently relayed back to the cortex for long-term storage during memory consolidation^72^. These processes are temporally separated and are modulated by acetylcholine (ACh). Studies suggest that ACh promotes memory encoding while suppressing the consolidation process^79^. Notably, ACh levels in the hippocampus rise during memory encoding but are low during memory consolidation^80^.

We observed no effect of varenicline at the doses tested in NOR. In previous rodent studies, varenicline has been shown to exert beneficial cognitive effects in the NOR task. For instance, chronic oral administration of varenicline at a dose of 3 mg/kg/day prevented memory impairment in the scopolamine mouse model (C57BL/6 male mice). Although varenicline effectively blocked scopolamine-induced memory impairment, it did not enhance the discrimination Index with respect to controls, consistent with our findings^41^. However, in a rat model (male Sprague–Dawley) acute varenicline administration (0.32–3.2 mg/kg, s.c.) improved novel object performance significantly in NOR task^81^. In contrast, we did not detect improved performance with varenicline alone at any dose tested. This discrepancy may be attributable to interspecies differences in anatomy, physiology, kinetics, receptors, and signal transduction pathways^82^. More recently, intra-mPFC varenicline infusion has been shown to dose-dependently enhance recognition memory in mice. In the same study, varenicline at subcutaneous doses of 0.032 and 0.32 mg/kg 1 h prior to sample trial significantly increased DI measured 24 h later in C57BL/6 mice^83^. It has been shown that there are critical differences in the underlying memory processes and brain regions recruited between short and long delays in the novel object recognition paradigm^84^ though typically in rat studies these are of the order of minutes for short delays and 24 h plus for long delays, it could be that these intervals vary in mice. A key difference between the study of Esaki et al. and the current study is that we used one habituation session while they used two. Additionally, we administered varenicline 30 min prior to the sample trial as opposed to one hour prior. While this is speculative it is possible that shorter habituation may lead to greater anxiety and consequently greater memory impairment rendering it less amenable to reversal, though it should be noted that we used a lengthy pre-experimental habituation period, and our test arena was very similar to the home cage. Another possible explanation for our lack of effect of varenicline alone is that we may not have reached similar drug levels 30 min after administration as at 1 h. Both possibilities merit further investigation in future studies evaluating pro-cognitive potential of varenicline.

To the best of our knowledge, cognitive properties of tropisetron have not been tested in mice using the NOR paradigm. We did not observe effects of tropisetron at the doses tested. Previous NOR studies performed in rats have shown that tropisetron enhances recognition memory at doses between 0.1 and 1 mg/kg, equivalent to the doses used in this study^85^. Similar explanations may apply to our findings with tropisetron. It is possible that species differences, dose range or different brain circuitries underlying different variants and stages of the NOR paradigm used may explain varied findings. nAChRs play a key role in the regulation of synaptic plasticity, which is essential for learning and memory^86^. These receptors are widely expressed throughout the medial prefrontal cortex (mPFC) while basal forebrain cholinergic neurons densely innervate the hippocampus, mediating the formation of episodic and semantic memory^80^. It has been shown that hippocampus input to the mPFC is crucial for recognition memory^87^ and that prefrontal nAChRs may play distinctive roles in associative recognition memory. Specifically, α7 nAChRs have been shown to be critical for encoding and the induction of long-term potentiation of hippocampus-mPFC synapses. On the other hand, α4β2 nAChRs have been found to be essential for retrieval via effects on long-term depression^88^.

When administered in combination varenicline and tropisetron significantly enhanced NOR performance. The effect was quite clear, was seen at all doses tested and has not been reported previously. It suggests that a synergistic effect of combination of these drugs in novel object recognition memory warrants further investigation. While behavioural effects at all doses can reflect general arousal as the mediating factor in cognitive performance, this is unlikely here as the total sample phase investigation times of the objects did not differ between drug groups and controls. These times were also comparable to those seen on other experiments with varenicline and tropisetron where no effect was observed. Control performance in the combination NOR experiment was lower than in other experiments possibly providing a baseline that permitted more sensitive detection of enhanced performance. This remains a possibility. However, we have theoretically modelled the additive effect of drugs combined on DI (supplementary Fig. S3) and the combination appears to supersede a simple additive effect. This is also evident when the DI data are expressed as percentage of their respective controls (supplementary Fig. S4). Notwithstanding, even if varenicline and tropisetron each had pro-cognitive effects somehow undetected in our paradigm, our data confirm that their combination retains a pro-cognitive effect, an important conclusion for clinical applications.

It is of interest to note that Rollema et al. reported that in mice varenicline potentiates the antidepressant effect of sertraline in the forced-swim test in mice^89^. This effect was observed in two mouse strains, suggesting it was not strain specific. Augmentation of antidepressant action of amitriptyline by nicotinic antagonist mecamylamine has also been reported^90^ and this augmentation strategy has been applied clinically^91^.

Both varenicline and tropisetron have been investigated independently for effects on cognition. Several studies have shown that single dose tropisetron improves cognitive performance in patients with schizophrenia^44–46^. Preclinical and clinical evidence supports varenicline’s potential to treat cognitive impairments in schizophrenia^16,68,69^. One impediment to development of these drugs for cognition has been gastrointestinal side-effects, tropisetron constipation due to 5HT3 antagonism and varenicline nausea thought to involve 5-HT3 agonism. By combining these drugs, it may be possible to offset these effects. This possibility merits further clinical investigation.

In conclusion we provide evidence consistent with previous findings α7 nAChR partial agonists potentiate latent inhibition. We further suggest that a potential synergism between tropisetron and varenicline in object recognition memory merits further investigation.

## Materials and methods

### Animals

Latent inhibition and NOR experiments were performed using C57BL/6 adult male mice (9–10 weeks old, 25–35 g) provided by Charles River, Kent UK. All mice were firstly acclimated upon arrival for 12 days and housed in standard cages under 12 h light-dark cycle, at 20–24 °C and humidity (40–60%). Health checks and 1 min-handling was performed from arrival of mice every day before the testing day. For latent inhibition test 120 animals were used per experiment (*n* = 15 per treatment). Animals were housed in separate cages (*n* = 3–4 per cage) with food and water available ad libitum during acclimatisation. Then, the animals were placed on a 22 h water restriction schedule 7 days prior to lick training and 23 h schedule throughout testing. A bottle of water was made available in each home cage for 1 h after each experimental session. For NOR test a total of 60 animals was used per experiment (*n* = 13–15 per treatment), housed in separate cages (*n* = 3–4 per cage) with food and water available ad libitum throughout acclimation and testing. Euthanasia method following testing was increasing concentration CO_2_. All experiments were carried out in accordance with the U.K. Home Office regulations on animal experimentation, with protocols approved by University of Nottingham Animal welfare Ethics Board with appropriate personal and project license authority under the Animals (Scientific Procedures) Act, UK 1986. UK Home Office Project License No: P16F842EF. Authors confirm reporting is in accordance with ARRIVE guidelines.

### Equipment

#### Novel object recognition

NOR experiments were conducted in an open-field arena (24.5 × 18.0 × 15 cm). This was positioned inside a soundproof cabinet containing an integrated overhead camera (video home-cage Med-Associates Inc, USA). During habituation, training, and test sessions, animals were recorded on the video camera positioned over the arena. Objects were made of glass/plastic and could be easily climbed on by mice (see supplementary Fig. S4 for examples). Objects were fixed to the arena with blue tack to secure them during exploration. This was changed between mice to prevent odour trails. Several copies of each object were used interchangeably and cleaned thoroughly with 1% cider vinegar to ensure the absence of olfactory cues. Videos were hand-scored by the trained experimenter who was blind to treatment groups. The experimenter was initially trained by an experienced colleague on a separate video of an unrelated experiment and permitted to score independently once scores were correlated at 0.95. Randomly selected video samples were cross-checked by a third experimenter blind to treatment for quality control purposes. Total activity counts were tracked from video using Ethovision XT17 (Noldus).

### Procedures

#### Latent inhibition

LI protocol was a described previously^65^. Briefly it consisted of five phases performed over 18 consecutive days: water restriction habituation, pretraining, pre-exposure, conditioning, reshaping/rebaseline and testing. (See also Table 1 for overview of experimental phases). Mouse is In Test the tone is presented and cessation of drinking in response to the tone is used as an index of learning. For latent inhibition to occur.

**Table 1:**
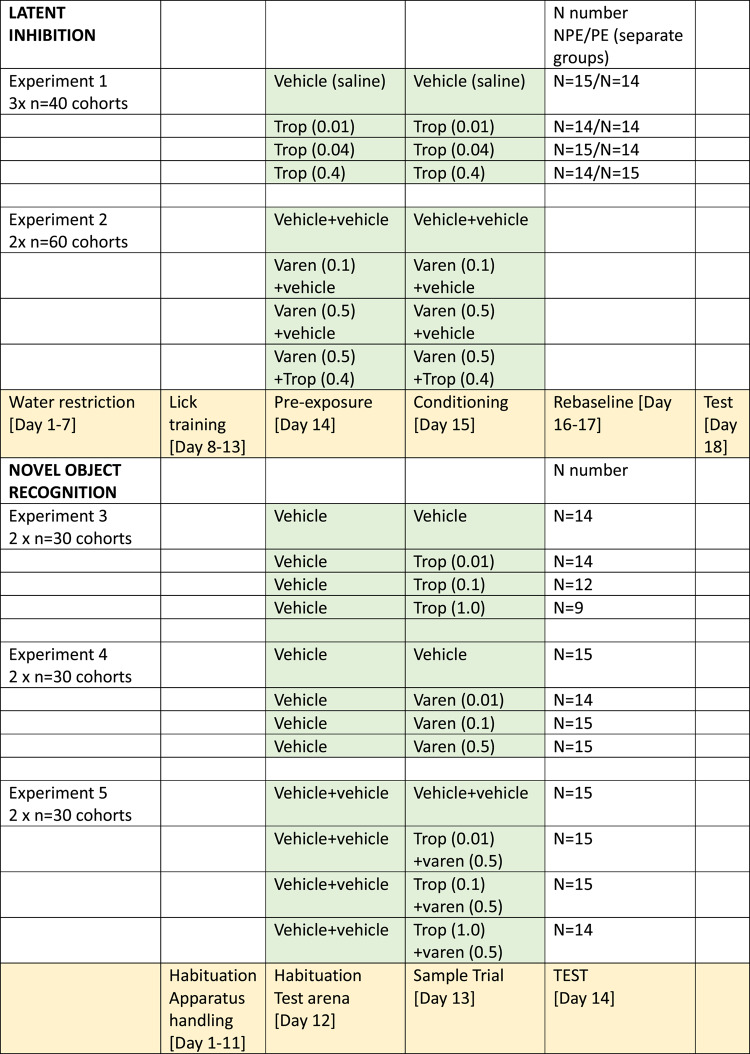
Experimental protocol (shaded yellow) and treatment groups (shaded green). Mice were injected prior to pre-exposure and conditioning in latent inhibition and habituation and sample trial stages of experiment in novel object recognition. Trop = tropisetron, varen=varenicline. Doses are mg/kg/s.c.

*Water restriction (Days 1–7): *Mice were placed on 22-hour water restriction 7 days before the experiment and maintained on 23-hour restriction throughout the experiment.

*Pretraining (Days 8–13): *This establishes stable baseline drinking behaviour. Mice were placed in skinner boxes for 15 min with water available in the box and the number of licks was recorded.

*Pre-exposure (Day 14)*: In pre-exposure mice are exposed to the tone or not and learn that it has no consequence. Mice were placed in skinner boxes without access to water in the box. The pre-exposure group (PE) received 60 of a 5-s 85-dB tone with an interstimulus interval of 15 s. Non pre-exposure (NPE) mice were placed in the skinner boxes for the same amount of time but received no tone presentations.

*Conditioning (Day 15)*:In conditioning all mice (both pre-exposed and non pre-exposed) learn an association between footshock and tone. Mice were placed in chambers without access to water in the box. After 5 min, two tone–footshock pairings were presented. Each tone (conditioned stimulus) was of 5-s duration and followed by a 1-s 0.38-mA footshock and an intertrial interval of 2.5 min. Mice remained in the chamber for 5 min following the second shock presentation.

*Reshaping/rebaseline drinking (Days 16 and 17)*: This re-establishes stable baseline drinking behaviour following conditioning to reduce contextual conditioning confounds. Mice were placed in skinner boxes for 15 min with water available and given free access to the water sipper to re-establish licking. Data from mice that did not resume drinking could not be analysed statistically for the Test-day (Day 18) analysis. These mice were however re-tested on a subsequent extinction day and included in supplementary analyses (Supplementary Figs. 1 & 2).

*Test (Day 18)*: In Test the strength of learning about the tone-footshock association established in conditioning is measured as cessation/slowing of drinking in response to the tone. Mice were placed in skinner boxes with water available and free access to the water sipper. The number of licks was recorded, and time taken to complete licks 80–90 (time A) and 90–100 (time B) recorded. After completion of 90 licks, the tone was presented until the mouse reached lick 100. Time B latencies were log transformed to permit parametric ANOVA analysis^60^. Higher log times indicate better learning. Latent Inhibition was defined as higher log_10_ time B latency in non-pre-exposed groups (NPE) compared to pre-exposed (PE).

### Novel object recognition

Following 11 days of handling and habituation to being placed in a test cage (see Table 1) the NOR experiment consisted of three sessions performed over three consecutive days; habituation, sample trial and test trial. Animals were habituated to the laboratory room 2 h before each session.

*Habituation*: animals were placed in an empty arena and allowed to explore freely for 5 min.

*Sample trial*: Mice were placed in an empty arena for 2 min, after which two identical objects were introduced. Mice were allowed to explore them freely for 5 min. Approach nose first directed towards the object, within 2–4 cm of the object was scored as object exploration. Behaviours such as climbing on the objects, using the objects as a platform to attempt escape from the arena etc. were not counted.

*Test trial*: mice were placed in an empty arena for two min after which two different objects were introduced (one identical to an object seen before, one novel) in the arena allowing the mice to explore them freely for 10 min.

NOR was measured as total investigation time of novel and familiar objects and a discrimination index. Total investigation time was calculated as time investigating novel object (s)/ time investigating novel object (s) + time investigating familiar object (s) * 100. Discrimination index (DI) was calculated as time investigating a novel object - time investigating familiar object (s) / time investigating novel (s) + time investigating familiar object (s). If total exploration time of both objects summed was less than 20 s, data was discarded for determination of total investigation time and discrimination index.

The object roles i.e., which object was allocated as familiar and which was allocated as novel, their relative positions in the arena, and treatments were counterbalanced and randomly assigned for each animal (see Supplementary Fig. 5). Randomisation of animals into treatment groups was performed using a computer-assisted method to ensure unbiased allocation. Each mouse was first identified by a unique ID and colour-coded label, then assigned a random number generated in Excel. These random numbers were used to rank the animals and assign them to each of the treatment groups. The assignment also included objects and their spatial location combinations to prevent any spatial or order bias such that objects were counterbalanced in terms of whether they were novel or familiar, and their spatial location in the arena. Exploration was defined as directed sniffing or intentional touching the object with either the nose or forepaws. Sitting/climbing on the objects was not considered exploratory behaviour.

### Pharmacological treatments

Tropisetron and varenicline were obtained from Tocris Bioscience, Bristol UK. Drugs were dissolved in 0.9% saline and subcutaneous injections (s.c.) administered in a volume of 1 ml/kg. All experiments were conducted blind to drug treatment. The drug solutions in identical vials were randomly re-labelled alphabetically for the experiment by a colleague not involved in the experiment and the code stored securely. Experiments were unblinded on completion of statistical analysis.

In latent inhibition experiments 1 and 2 a single injection of either drug or saline treatment was administered subcutaneously 15 min prior to both the preexposure phase (day 14) and conditioning phase (day 15). Vehicle control groups received saline solution. In experiment 1 investigating the effects of tropisetron doses of 0.01, 0.04 or 0.4 mg/kg were administered. In experiment 2 investigating the effect of varenicline and tropisetron on LI, doses of 0.1 and 0.5 mg/kg of varenicline alone; and combined 0.5 mg/kg varenicline and 0.4 mg/kg of tropisetron were administered. Doses of varenicline were selected based on a compromise range of doses shown to improve cognition in patients when given multiple times daily chronically^68^. Doses of tropisetron were selected as dose equivalents that show improved cognition and anti-nausea effects in patients^46,52^. Doses were converted from human oral to mouse subcutaneous doses using standard m^2^ body surface area conversion formulae^92^.

Each NOR experiment was performed in two cohorts of 30 mice on two separate weeks following 11 days of habituation to the apparatus. Mice were given one subcutaneous injection of sterile saline 30 min prior to habituation on day 1 to habituate them to the injection procedure. In experiments 3–5, drugs were administered prior to sample phase. In experiments investigating the effect of tropisetron (experiment 3) or varenicline (experiment 4) on NOR mice received one subcutaneous injection of saline 30 min prior to drug injection on the sample trial (see Table 1). This was to match them for two administrations which would be necessary for upcoming experiment 5 combining both drugs. 20 min prior to the sample trial mice received either tropisetron experiment 3 (0.01 mg/kg, 0.1 mg/kg or 1 mg/kg) varenicline experiment 4 (0.01 mg/kg, 0.1 mg/kg and 0.5 mg/kg) or saline. Experiment 5 investigated the effect of combined tropisetron and varenicline, four experimental groups were tested: saline control; tropisetron (0.01 mg/kg) + varenicline (0.5 mg/kg); tropisetron (0.1 mg/Kg) + varenicline (0.5 mg/kg) and tropisetron (1 mg/kg) + varenicline (0.5 mg/kg). Doses of varenicline and tropisetron were selected based on a pro-cognitive effect in experiment 2 (varenicline) and a clinically relevant dose (tropisetron). Subcutaneous injection of tropisetron and varenicline were administered 30 and 20 min respectively prior to sample trial. Pretreatment times were selected to match as closely as possible those in the prior latent inhibition experiment. See Table 1 for details on treatment groups and experimental procedure.

### Experimental design and statistics

#### Latent inhibition

Experiment 1 (tropisetron) was conducted in three cohorts of 40 mice (*n* = 120). Half of the animals in each cohort were assigned to 60 pre-exposures (PE) of the tone stimulus, while the other half received no pre-exposure (NPE). Due to experimental equipment error the first five mice data (1 session) from cohort 1 were not recorded (1 x saline PE,1 tropisetron 0.01 NPE,1 tropisetron 0.01 PE, 1 tropisetron 0.04 PE, 1 tropisetron 0.4 NPE) resulting in *n* = 115 for statistical analysis.

Experiment 2 (varenicline/ varenicline +tropisetron) was conducted in two cohorts of 60 mice (*n* = 120) (see Table 1). Half of the animals in each cohort were assigned to 60 pre-exposures of the tone stimulus, while the other half received no pre-exposure. Eight treatment groups were tested (see Table 1 for sample sizes). Eight mice did not resume drinking after rebaseline training (1 saline NPE, 1 saline PE, 1 tropisetron 0.1 NPE, 2 tropisetron 0.1 PE, 1 tropisetron 0.5 NPE, 2 combination PE) and were excluded from the analysis. Four of these mice resumed drinking on test day 2 thus their data were available for analysis of test day 2 data reported as supplementary data.

Statistics were performed using GraphPad Prism (Version 9.4.0 (673) and JASP (version 0.16.3/4 (JASP Team (2022). JASP (Version 0.16.3)[Computer software]. For both LI experiments, analysis of variance (ANOVA) was used with Exposure (NPE/PE) and Drug (doses as per experiment)as factors. Levene’s test confirmed ANOVA normality assumptions Experiment 1 F(7,107) = 0.564, *P* = 0.7 / Experiment 2 F(7,104) = 0.9, *P* = 0.50). Post hoc comparisons (NPE Vs PE) were performed using T-tests with Bonferroni correction for family-wise error rate.

### Novel object recognition

Experiments 3, 4 and 5 were conducted each in two cohorts of 30 mice (*n* = 60) (see Table 1). Statistics were performed using GraphPad Prism (Version 9.4.0 (673). For all NOR experiments, data were analysed using one-way ANOVA with Drug Group factor following Brown-Forsythe normality assumption tests ; Experiment 3 F(3,45) = 0.23, *P* = 0.8) Experiment 4 F(3,55) = 1,67 *P* = 0.18), Experiment 5 F(3,55) = 3.2, *P* = 0.02). Dunnett’s post hoc tests were used to compare each treatment group to the saline vehicle control. Experiment 3 used an arena that had a small grill blocked off to prevent mice from investigating it. During video analysis it was observed that several mice spent a significant amount of time perseveratively investigating the grill (a behaviour that did not occur in our set up experiments). As these exploration times could not be considered as accurate they were excluded from the statistical analysis (*N* = 1 vehicle, *N* = 2 Trop 0.1,*N* = 5 Trop (1.0) (which was carried out blind to treatment). We used an identical arena without this grill for subsequent experiments. We also excluded mice that did not spend at least 20 s in total investigating the objects (*N* = 3 exp1, *N* = 1 exp 4, *N* = 1 exp 5).

#### Latent inhibition

All LI experiments were carried out in five identical skinner boxes located within a sound-attenuating cabinet. The skinner boxes (conditioning chambers) had plexiglass walls at the front and back, stainless steel sides, and a metal grid floor connected to a shock scrambler and generator. Each box contained a ventilation fan, mounted at the back to provide air exchange and background noise (69 dB white noise), and a sonalert mounted on the right wall for delivering the conditioned stimulus tone (85 dB) (ENV-323AW, Med Associates Inc., St. Albans, VT, USA). Each chamber was equipped with a removable drink spout located in the left wall of the chamber. The lick spout was connected to a lickometer (ENV-250, Med Associates Inc., St. Albans, VT, USA) which recorded the number of licks. The skinner boxes were interfaced with a PC computer running MED-PC software (SOF-735, MED Associates Inc., St. Albans, VT, USA) to control stimulus presentation and record data.

## Supplementary Information

Below is the link to the electronic supplementary material.

Supplementary Material 1

Supplementary Material 2

Supplementary Material 3

Supplementary Material 4

Supplementary Material 5

Supplementary Material 6

## Data Availability

The datasets used and/or analysed during the current study are available from the final author on reasonable request.
