# Supplementary figures and images for: Investigation of varenicline and tropisetron in latent inhibition and novel object recognition in mice
Source: Sci Rep. 2026 Mar 3;16:11823. doi: 10.1038/s41598-026-41544-w (PMC13066101; doi:10.1038/s41598-026-41544-w)

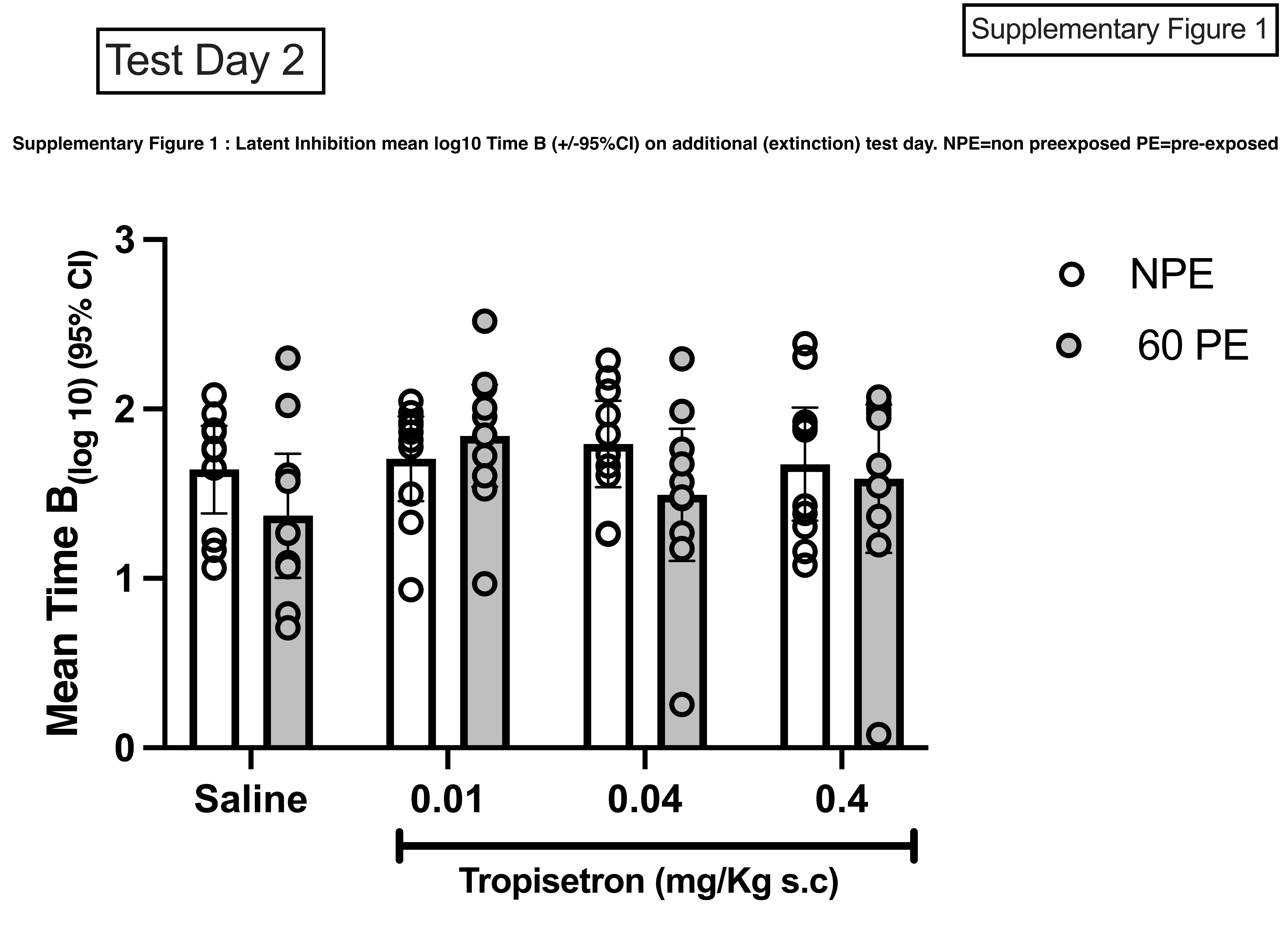

Supplement: Supplementary file 1 — Supplementary Material 1 [file 41598_2026_41544_MOESM1_ESM.tiff]

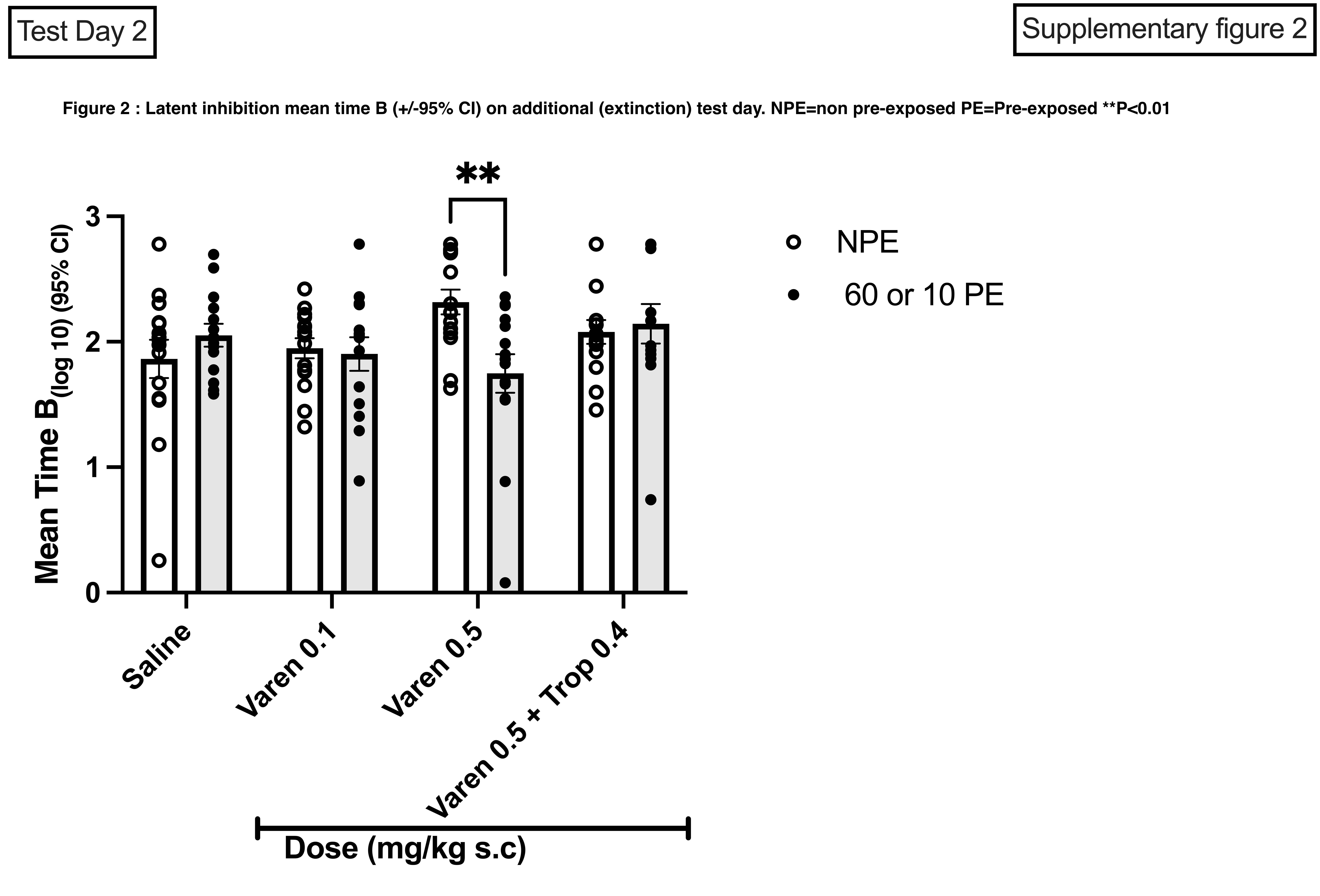

Supplement: Supplementary file 2 — Supplementary Material 2 [file 41598_2026_41544_MOESM2_ESM.tiff]

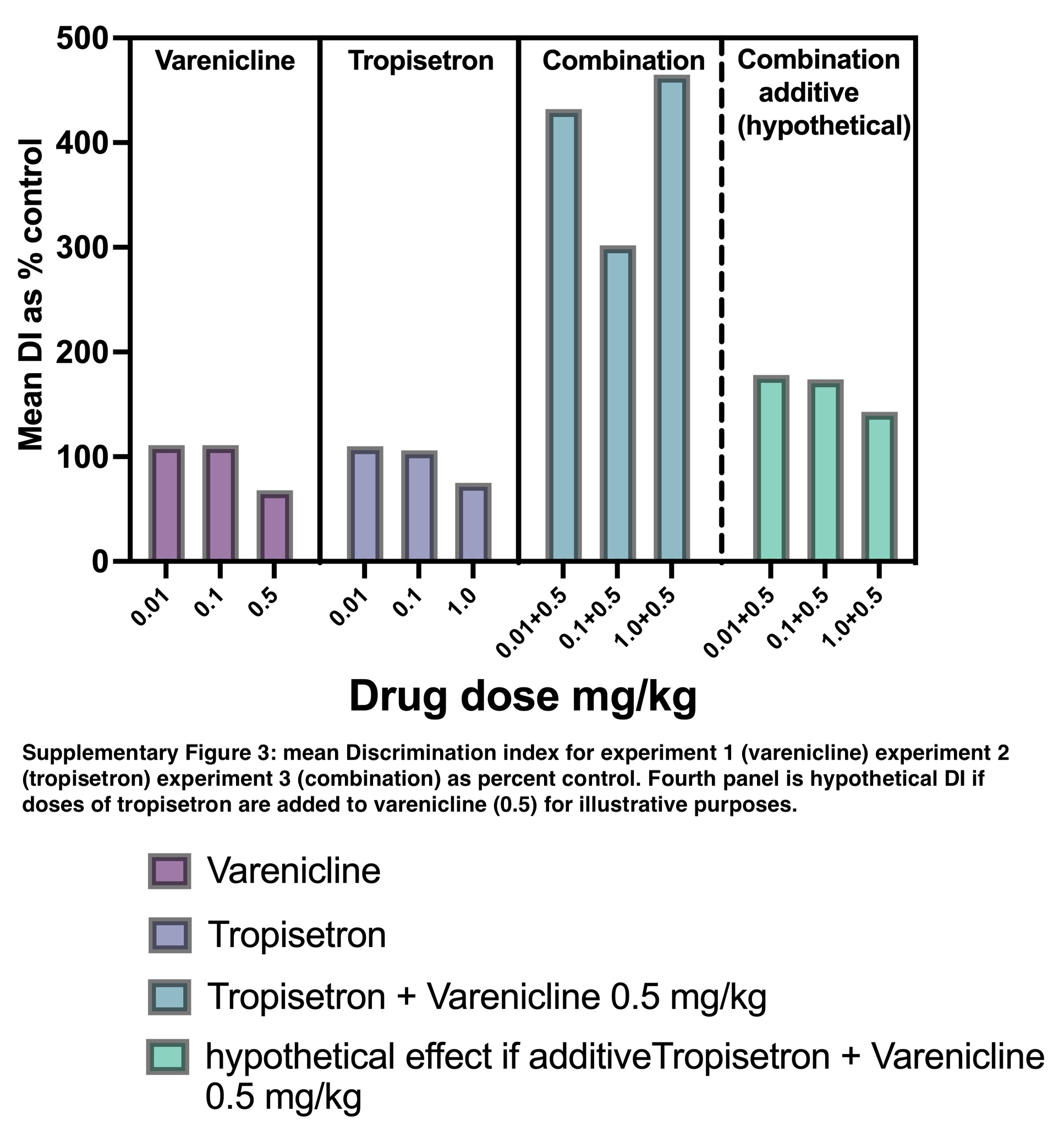

Supplement: Supplementary file 3 — Supplementary Material 3 [file 41598_2026_41544_MOESM3_ESM.tiff]

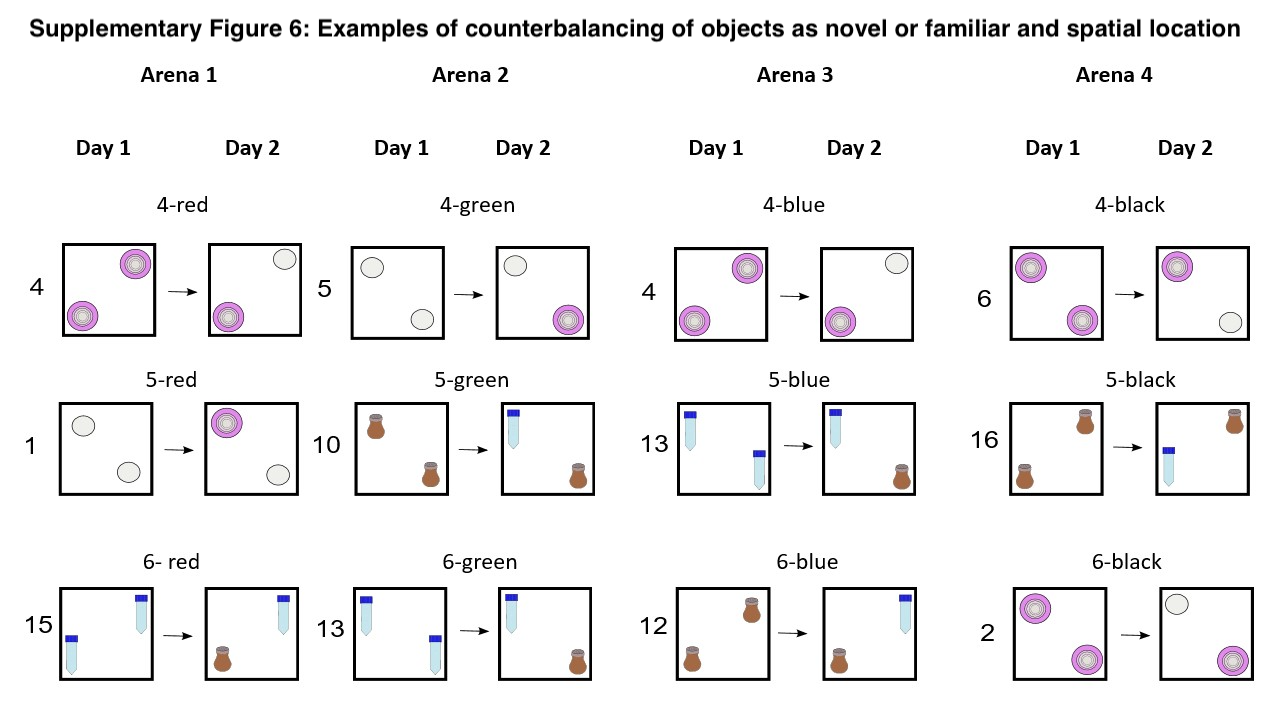

Supplement: Supplementary file 5 — Supplementary Material 5 [file 41598_2026_41544_MOESM5_ESM.tiff]

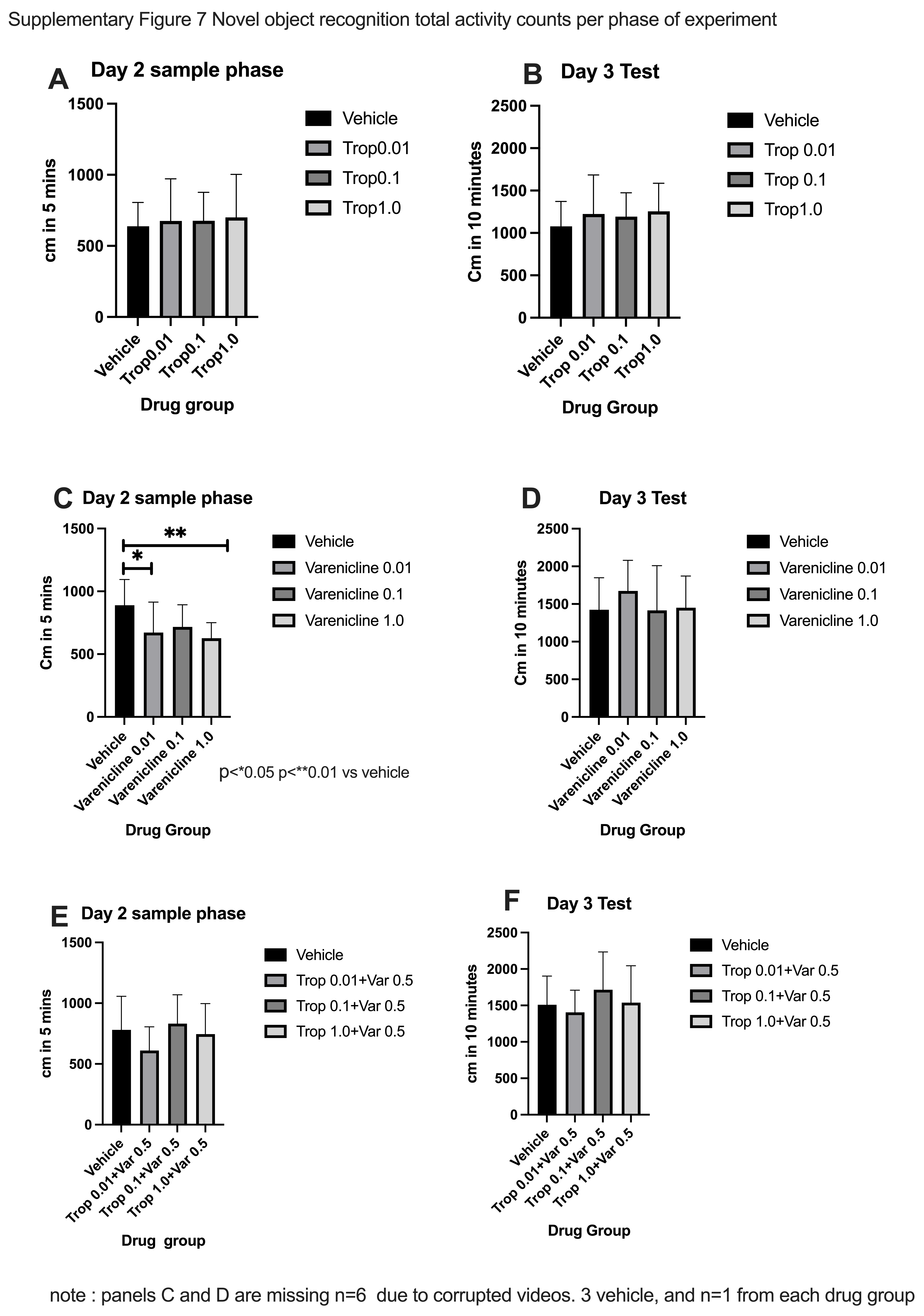

Supplement: Supplementary file 6 — Supplementary Material 6 [file 41598_2026_41544_MOESM6_ESM.tiff]
