# Supplementary material for: Investigation of varenicline and tropisetron in latent inhibition and novel object recognition in mice
Source: Sci Rep. 2026 Mar 3;16:11823. doi: 10.1038/s41598-026-41544-w (PMC13066101; doi:10.1038/s41598-026-41544-w)
Supplement: Supplementary file 4 — Supplementary Material 4 [file 41598_2026_41544_MOESM4_ESM.docx]

**Supplementary Figure 5 :**  Examples of objects used for NOR experiments. During test objects were temporarily fixed to the arena to prevent being knocked over during exploration. Scale bar = 4 cm
